# Investigating the Applicability of Alignment—A Monte Carlo Simulation Study

**DOI:** 10.3389/fpsyg.2022.845721

**Published:** 2022-06-24

**Authors:** Congcong Wen, Feng Hu

**Affiliations:** ^1^International College, Xiamen University, Xiamen, China; ^2^Institute of Education, Xiamen University, Xiamen, China; ^3^Xiamen National Accounting Institute, Xiamen, China

**Keywords:** Monte Carlo simulation study, multiple-group analysis, alignment, measurement invariance, cross-loading

## Abstract

Traditional multiple-group confirmatory factor analysis (multiple-group CFA) is usually criticized for having too restrictive model assumption, namely the scalar measurement invariance. The new multiple-group analysis methodology, alignment, has become an effective alternative. The alignment evaluates measurement invariance and more importantly, permits factor mean comparisons without requiring scalar invariance which is usually required in traditional multiple-group CFA. Some simulation studies and empirical studies have investigated the applicability of alignment under different conditions, but some areas remain unexplored. Based on the simulation studies of Asparouhov and Muthén and of Flake and McCoach, this current simulation study is broken into two sections. The first study investigates the minimal group sizes required for alignment in three-factor models. The second study compares the performance of multiple-group CFA, multiple-group exploratory structural equation model (multiple-group ESEM), and alignment by including different proportions and magnitudes of cross-loadings in the models. Study 1 shows that when the model has no noninvariant parameters, the alignment requires relatively lower group sizes. Explicitly, the minimal group size required for alignment was 250 when the amount of groups was three, the minimal group size was 150 when the amount of groups was nine, and 200 when the amount of groups was 15. When there are noninvariant parameters in the model and the amount of groups is low, a group size of 350 is a safe rule of thumb. When there are noninvariant parameters in the model and the amount of groups is high, a group size of 250 is required for trustworthy results. The magnitude of noninvariance and the noninvariance rate do not affect the minimal group size required for alignment. Study 2 shows that multiple-group CFA provides accurate factor mean estimates when each factor had 20% factor loading (1 factor loading) with small-sized cross-loading. Multiple-group ESEM provides accurate factor mean estimates when the magnitude of cross-loading is small or when each factor had 20% factor loading (1 factor loading) with medium-sized cross-loading. Alignment provides accurate factor mean estimates when there are only small-sized cross-loadings in the model. The parameter estimates, coverage rates and ratios of average standard error to standard deviation for each methodology are not influenced by the amount of groups. Recommendations are concluded for using multiple-group CFA, multiple-group ESEM, traditional alignment and aligned ESEM (AESEM) based on the results. Multiple-group CFA is more suitable for use when scalar invariance is established. Multiple-group ESEM works best when there are small-sized or only a few medium-sized cross-loadings in the model. Traditional alignment allows for small-sized cross-loadings and a few noninvariant parameters in the model. AESEM integrates the advantages of alignment and ESEM, can provide accurate estimates when noninvariant parameters and cross-loadings both exist in the model. Compared to multiple-group CFA, multiple-group ESEM, the alignment methodology performs well in more situations.

## Introduction

In most empirical studies, researchers are generally not content to only investigate the structure of an instrument, but also take an interest in comparing the factor scores or factor means of different subgroups *via* some background variables, such as gender, nationality, education level, and socio-economic status. In order to do this, researchers often use multiple-group confirmatory factor analysis (multiple-group CFA) to make such group comparisons.

It is well known that the establishment of measurement invariance across groups is a prerequisite for conducting substantive cross-group comparisons. For unobserved factors with multiple observed indicators, measurement invariance can be tested using multiple-group CFA. Measurement invariance involves four different levels: configural invariance, weak measurement invariance (also called metric invariance), strong measurement invariance (also called scalar invariance), and strict measurement invariance (Meredith, 1993; Widaman and Reise, 1997; Millsap, 2012; Caycho-Rodríguez et al., 2021). Configural measurement invariance is when there are the same number of factors and the same patterns of free and fixed factor loadings across groups without equality restrictions on any other model parameters. Weak measurement invariance, also called metric invariance, is defined as an invariance of factor loadings across groups, indicating that the factor loadings for the different groups are statistically equal. Strong measurement invariance, also called scalar invariance, is defined as invariance of both factor loadings and item intercepts across groups. In general, when strong measurement invariance holds, the factor means across groups can be compared directly (Millsap, 2012; Caycho-Rodríguez et al., 2021).

The assumption of scalar invariance in multiple-group CFA has long been strongly criticized because this assumption is too restrictive to be established. In real data sets, such as in cross-national PISA research involving many groups, factors, indicators, and participants, an acceptable fit of the complete scalar invariance model is rarely achieved (Davidov et al., 2014; Nagengast and Marsh, 2014; Rutkowski and Svetina, 2014; He and Kubacka, 2015; Zercher et al., 2015). Therefore, direct group means comparison is not feasible in this context (Wen et al., 2019). When metric invariance holds but scalar invariance fails, researchers usually use a forward stepwise selection process to achieve an acceptably fitted partial scalar invariance model. This process is done by relaxing the parameters with the largest modification indices one by one, until the freeing of additional parameters no longer offers substantial improvement to the model fit. One issue is that, when the amount of groups is large or the model is quite complex, this stepwise strategy may require many modifications, making this process particularly cumbersome. The acceptably fitted model exploration accomplished by relaxing the parameters with the largest modification indices of the scalar invariance model could well lead to the wrong model because the scalar invariance model itself is far from the true model (Asparouhov and Muthén, 2014). When the observed indicators predicting the outcome variables have high correlations, the multicollinearity may exist in the modification indices (Marsh et al., 2018). The better fitted partial scalar invariance model does not guarantee the accuracy of the specific factor mean estimates in the exact area of research interest.

To address these issues, Asparouhov and Muthén proposed a new methodology called alignment in 2014. The alignment methodology can be used to estimate group-specific factor means and variances without requiring exact measurement invariance (Muthén and Asparouhov, 2014). Specifically, the alignment tests the approximate measurement invariance of the model in order that the factor mean comparison is meaningful. Approximate measurement invariance allows small differences in measurement parameters assuming such small differences do not affect the results of subsequent group mean comparisons (Kim et al., 2017). While even just a few noninvariant parameters make the group mean comparisons infeasible in multiple-group CFA, alignment can provide trust worthy factor mean and measurement parameter estimates by establishing the approximate measurement invariance of the model (Lomazzi, 2018; Munck et al., 2018). This methodology also provides a detailed account of parameter invariance for every model parameter in every group (Muthén and Asparouhov, 2014).

## Model Formulation

The alignment methodology was first proposed by Asparouhov and Muthén, 2014. It is a methodology based on multiple-group CFA. Before going to the literature review section, readers may expect to know what alignment is and how the alignment optimization proceeds. At first, a brief introduction of the alignment optimization should be presented.

Consider the multiple-group CFA model:


(1) $$\;y_{ipg}=v_{pg}+\overset{m}{\underset{m=1}{\sum }}\lambda_{pmg}\eta_{img}+\varepsilon_{ipg}$$

where *y* is the observed indicator, *v* is the intercept, *λ* is the factor loading, *η* is the factor, and *ε* is the residual. Because these parameters belong to different groups, factors, indicators, or cases in specific groups, subscripts are used. Specifically, where *p* = 1, 2, …, *p* represents the ordinal number of the observed indicators; where *m* = 1, 2, …, *m* represents the ordinal number of the factors; where *g* = 1, 2, …, *g* represents the ordinal number of the groups; where *i* = 1, 2, …, *i* represents the ordinal number of the independent case *i* in group *g*; and finally, we assume that ε _ipg~_*N* (0, *θ*_pg_), *η*_img~_*N* (*α*_g_, *ψ*_g_).

Different from multiple-group CFA, alignment is based on a configural invariance model to compare factor means across groups. Assuming that the configural invariance model is M0, the intercept and factor loading are denoted by *v*_pg0_ and *λ*_pg0_, respectively. The model M0 transforms the factor of each group to mean zero and variance one. This transformation allows the variance and mean of the indicators to be expressed as:


(2) $$V\;\left(y_{\mathrm{pg}}\right)=\lambda^{2}{}_{\mathrm{pmg}}\psi_{g}=\lambda^{2}{}_{\mathrm{pmg},0}$$

(3) $$E\;\left(y_{\mathrm{pg}}\right)=v_{\mathrm{pg}}+\lambda_{\mathrm{pmg}}\alpha_{g}=v_{\mathrm{pg},0}$$

For *α*_g_ and *ψ*_g_ of any group, there are corresponding intercept *v*_pg_ and factor loading *λ*_pmg_ that yield the same likelihood as the configural model M0. The *λ*_pmg_ and *v*_pg_ in Equations (2) and (3) can be calculated *via* these two equations because the *λ*_pmg,0_ and *v*_pg,0_ of the configural model are already known. The corresponding intercepts and loadings are chosen such that the measurement noninvariance between groups is minimized. This is done with respect to *α*_g_ and *ψ*_g_ using a loss/simplicity function, F, that accumulates the total measurement noninvariance:


(4) $$\begin{array}{c} \;F=\underset{p}{\sum }\underset{g1<g2}{\sum }W_{g1,g2}f\left(\lambda_{pmg1}-\lambda_{pmg2}\right)\\ +\underset{p}{\sum }\underset{g1<g2}{\sum }W_{g1,g2}f\left(v_{pmg1}-v_{pmg2}\right) \end{array}$$

For every pair of groups’ intercepts and loadings, the differences between the parameters are accumulated and then scaled by *f*, the component loss function (CLF). The groups are weighted by their sample size, *W*, such that larger groups will contribute more to the total loss function, *F*. In alignment, the CLF is:


(5) $$f\left(x\right)=\sqrt{\sqrt{x^{2}+\varepsilon }}$$

The CLF has also been used in exploratory factor analysis (EFA) rotation algorithms to estimate factor loadings with the simplest possible structure (Jennrich, 2006). Asparouhov and Muthén (2014) have described the baseline model, M0, and the resulting model, M1, as paralleling the unrotated and rotated factor solutions in EFA. The total loss function is minimized at a solution where there are few large noninvariant parameters and many approximate invariant parameters.

Minimizing the total loss function will generally identify the parameters *α*_g_ and *ψ*_g_ in all groups except the first. In free alignment, *α*_1_ is estimated as a free parameter, while in fixed alignment, *α*_1_ is set to 0 although this constraint is generally unnecessary. In order to calculate the last factor mean in free alignment, we use the following parameter constraints:


(6) $$\psi_{1}\times \psi_{2}\times \ldots \times \psi_{g}=1$$

## Literature Review

Cross-cultural and/or national empirical studies usually involve factor mean comparisons and scalar invariance is seldom achieved with multiple-group CFA (Byrne and van de Vijver, 2010; Davidov et al., 2012; Meuleman and Billiet, 2012; Oberski, 2014). As a new statistical methodology, alignment has been drawing attention in recent years. Alignment has become a viable alternative as it has the ability of estimating a large amount of groups and quite a few noninvariant parameters.

For example, Munck et al. applied the alignment methodology in the analysis of adolescents’ support for immigrant rights in a pooled data set taken from the 1999 International Association for the Evaluation of Educational Achievement (IEA) Civic Education Study and the 2009 International Association for the Evaluation of Educational Achievement (IEA) International Civics and Citizenship Education Study. Measurement invariance was examined across 92 groups (country by cohort and by gender) of a five item-one factor instrument with a Likert-type scale. The data was gathered from a total of 79,278 subjects, with an average group size of 862. They found that the frequentist alignment, which used maximum likelihood estimation, made it feasible to comprehensively assess measurement invariance in large data sets and to compute aligned factor scores for the full sample in order to update existing databases for further, more efficient secondary analysis while incorporating metainformation concerning measurement invariance (Munck et al., 2018).

Lomazzi assessed the measurement equivalence of the Gender Role Attitudes Scale included in the last wave of the World Values Survey distributed across 59 countries. The scale had five items measuring one factor, with a Likert-type response scale. The data had 89,320 subjects in total, and the average group size was 1,514. Using the frequentist alignment methodology, an acceptable degree of non-invariance was achieved for 34 countries. The author confirmed that the frequentist alignment procedure is a viable alternative to the multiple-group CFA. Alignment maintained good model fit in the most convenient model and allowed factor mean comparisons for a large amount of groups (Lomazzi, 2018).

Besides the applications in empirical studies, Monte Carlo Simulation Study is one of the best ways to investigate the applicability and effectiveness of alignment under different conditions. At the time of this study, six simulation studies regarding the alignment methodology have been published. Table 1 shows the comparison of existing simulation studies about alignment.

**Table 1:** Comparison of existing simulation studies about alignment.

Study	Models	Estimator	Identification option	Design	Main conclusions
1. Asparouhov and Muthén, 2014	one factor, five continuous items, four amounts of groups (2, 3, 15, 60)	ML	Fixed alignment and free alignment	Three noninvariance rates (0, 10, 20%), two group sizes (100, 1,000)	When factor mean value generated in the reference group is 0, both fixed alignment and free alignment provide accurate estimates. Fixed alignment performs better than free alignment. Frequentist alignment needs large sample size to provide accurate estimates.
	One factor, five continuous items, 3 groups	ML, Bayes	Free alignment	Noninvariance rate is 20%, five group sizes (300, 1,000, 2,000, 5,000, 10,000)	The Bayes estimator gives slightly more accurate standard errors than ML estimator. ML standard errors are overestimated for small sample sizes.
	one factor, five continuous items, five amounts of groups (3, 5, 10, 15, 20)	ML	Fixed alignment and free alignment	Noninvariance rate is 20%, group size is 1,000	When factor mean value generated in the reference group is 1, the amount of groups is at least three, and noninvariance exists in the model, free alignment performs better than fixed alignment.
2. Kim et al., 2017	One factor, six continuous items, two amounts of groups (25, 50)	ML	Fixed alignment	Noninvariance rate is 33.3%, three group sizes (50, 100, 1,000)	Alignment appears more optimal under approximate invariance than substantial noninvariance for detecting invariant and noninvariant model parameters.
3. Flake and McCoach, 2018	Two factors, seven polytomous items per factor, four categories per item, three amounts of groups (3, 9, 15)	ML robust	Fixed alignment	Four noninvariance rates (0, 14, 28, 43%), three magnitudes of noninvariance (loading: small −0.1, medium −0.25, large −0.4; intercepts: small −0.2, medium −0.5, large −0.8), group size is 500	Alignment provides accurate parameter estimates under most conditions of small magnitudes of noninvariance and low noninvariance rates (14, 29%) when the noninvariant parameters are only factor loadings. Alignment get accurate intercept and factor mean estimates when the noninvariance rate is below 29%, the magnitude of noninvariance is medium or large, and the noninvariant parameters are only intercepts
4. Marsh et al., 2018	One factor, five continuous items, 15 groups	ML	Fixed alignment	Two noninvariance rates (10% large +90% small; 20% large +80% small), two magnitudes of noninvariance (loading: small 1 ± 0.05, 1 ± 0.1, large 1.4, 0.5, or 0.3; intercepts: small ±0.05, ±0.1, large ±0.5), two group sizes (100, 1,000),	Alignment outperforms both the complete and partial scalar approaches when there is no support for complete scalar invariance. Alignment performs no worse than complete scalar invariance model when there is support for complete scalar invariance. Cross-validation using new data from the same population generating model supports the superiority of alignment to both the complete and partial scalar invariance models.
5. Muthén and Asparouhov, 2018	One factor, four continuous items, 26 groups	ML, Bayes	Not mentioned	Use estimates based on the 26 European countries data as population values, compare the performance of alignment and the performance of two-level random-intercept random-slope model	Alignment outperforms two-level approach when the model has small amount of items, small amount of groups, the measurement parameters are non-normal. It is also available with complex survey data. While the two-level approach outperforms alignment when the model has more than 100 groups, small group sizes, weak invariance pattern, or when it’s necessary to relate non-invariance to other variables.
6. Pokropek et al., 2019	One factor, three amounts of items (3, 4, 5), 24 groups	ML	Fixed alignment	*N*−1 noninvariance rates (i.e., for *n* items, 1, 2 …*n*−1 noninvariant items conditions are considered), group size is 1,500	Alignment is recommended for recovering latent means in cases where there are only few noninvariant parameters (no more than 20% of the parameters).

In general, there is some similarity regarding the models chosen, estimation method and identification option, but some differences regarding the research design, research purpose and conclusion among these studies. These studies investigated the applicability of alignment under different conditions, compared the performance of alignment and the performance of other models. However, some important issues still need to be further explored. For example, in terms of the average group size, the majority of previous simulation studies have considered limited amounts of sample sizes (e.g., 100, 500, 1,000, 2000, 5,000, 10,000) and have not investigated the minimal group sizes required for alignment. The minimal group size required for alignment is an important issue with regard to its applicability. In this current study, one of the primary goals was to investigate the minimal group sizes required for alignment under different simulation conditions. Secondly, because neglecting the estimation of cross-loadings in multiple-group CFA can lead to substantial inflated estimates of the principal factor loadings, factor covariances (Asparouhov and Muthén, 2009) and alignment itself is based on multiple-group CFA model, its performance is best tested when cross-loadings are generated in the model but neglected in the estimation. No previous study has investigated the impact of cross-loadings on alignment. The current study attempts to investigate this issue and compare alignment with both multiple-group CFA and multiple-group ESEM. After presenting these comparisons, conclusions and recommendations on these three methodologies follow. Through this article, we hope to highlight how and when to use each of these three methodologies, especially alignment.

## Study 1: Minimal Group Size Required for Alignment

### Some Default and Manipulated Settings

Before presenting the design of the research, some issues should be addressed.

First, the fixed alignment is used as the identification methodology. According to the previous simulation studies, when the factor means of the reference group are equal to or slightly different from 0, fixed alignment performs better in almost all situations than free alignment. When the factor means of the reference group are significantly different from 0, this means that there are misspecifications in the model, leading to biased estimates of the parameters (Asparouhov and Muthén, 2014). When the factor means of the reference group are significantly different from 0, free alignment is better than fixed alignment when the amount of groups is larger than two and there is noninvariance (Asparouhov and Muthén, 2014). Due to the fact that main focuses of this current study are the noninvariance rate, the minimal group size required for alignment, and the accuracy of the parameter estimates when there are cross-loadings, the influence of different alignment identification options should be excluded. Comparing the performance of fixed alignment when the factor means of the reference group are 0 with the performance of free alignment when the factor means of the reference group are not 0, the estimates of the former fixed alignment are more accurate in Asparouhov and Muthén (2014) as well as in this current study. Therefore, the factor means of the first group (as the default reference group) are designed to be zero values and fixed alignment is chosen as the identification methodology.

Second, maximum likelihood estimation is used as the estimation method. Frequentist maximum likelihood estimation and Bayesian estimation are now available in Mplus alignment optimization. The Bayesian method does not rely on asymptotic theory and is more empirically driven, whereas the ML method relies on asymptotic theory but is independent of prior specifications (Asparouhov and Muthén, 2014). One advantage of BSEM alignment estimation over maximum likelihood alignment estimation is that the BSEM model allows small residual covariance between indicators, leading to a better model fit (Muthén and Asparouhov, 2012). A detailed explanation of the Bayesian approach for testing approximate measurement invariance is beyond the scope of this study, but can be found in the papers of Muthén and Asparouhov (2013, 2018) and Van de Schoot et al. (2013). In the current study, only the frequentist maximum likelihood alignment estimation is used in the simulation for the following reasons. First, in almost all empirical studies using alignment, the frequentist method is chosen instead of the Bayesian method (Weziak-Bialowolska, 2015; Byrne and van de Vijver, 2017; Jang et al., 2017; Żemojtel-Piotrowska et al., 2017; Lomazzi, 2018; Munck et al., 2018). As such, the frequentist method deserves more investigation. Second, the Bayesian method is based on previous theoretical applications where practitioners choose informative priors. However, in some studies, practitioners may only know a limited amount of information about the instrument and the data. From this perspective, investigation of the frequentist method is more valuable.

Third, the current study attempted to investigate the performance of alignment in three-factor models. This is because the complexity of three-factor models is quite reasonable and very common when analyzing actual complex survey data (Ali et al., 2018; Rajalingam et al., 2018; Pérez-Fuentes et al., 2019; Torff and Kimmons, 2021). Hence, the results of the current study may be generalizable.

Before demonstrating the simulation design, we could compare the simulation conditions of them. The details of the simulation conditions are summarized in Table 2.

**Table 2:** Summary of the simulation conditions of the two simulation studies.

Manipulated conditions	Study 1	Study 2
Models estimated	Alignment	Multiple-group CFA, Multiple-group ESEM, alignment
Amount of groups	3, 9, 15	3, 9, 15
Average group size	Start from 100	1,000 per group
Noninvariance rates	0, 10, 20%	0%
Magnitudes of noninvariance	Small, large	—
Type of noninvariance	Loadings and intercepts mixed	—
Locations of noninvariant parameters	Noninvariant loading and intercept not on same indicator	—
Rates of indicators having cross-loading	—	20, 40%
Magnitudes of Cross-loadings	—	Small, medium
*Constant conditions*		
Factor distributions	Three types, *N* ~ (0, 1), *N* ~ (0.3, 1.5), *N* ~ (1, 1.2)	Three types, *N* ~ (0, 1), *N* ~ (0.3, 1.5), *N* ~ (1, 1.2)
Factor model	Three factors, each factor has five continuous indicators	Three factors, each factor has five continuous indicators
Estimator	Maximum Likelihood	Maximum Likelihood
Identification option	Fixed	Fixed

### Research Design of Study 1

The first study attempted to investigate the minimal group sizes required for alignment when the amount of groups, magnitude of noninvariance, and noninvariance rate are different. The fitted alignment model was based on configural invariance. The current study used three factor-models as many empirical studies investigate this number of factors (Ali et al., 2018; Rajalingam et al., 2018; Pérez-Fuentes et al., 2019; Torff and Kimmons, 2021). Each factor included five continuous indicators as this is quite practical in real data (DiStefano, 2002; Beauducel and Herzberg, 2006; Muthén, 2006; Clark, 2010; Asparouhov and Muthén, 2014; Munck et al., 2018; Pokropek et al., 2019). The initial invariant factor loadings were 0.8 (For unstandardized invariant factor loadings, the standardized values were 0.63 for the first group type, 0.70 for the second group type, 0.66 for the third group type.), invariant intercepts were 0, residual variances were 1, factor covariances were 0.5 (Standardized factor correlations were 0.5 for the first group type, 0.33 for the second group type, 0.42 for the third group type.). These values are in line with the study of Asparouhov and Muthén (2014) and the results of the current study may be compared with the results of their study. We manipulated three amounts of groups (i.e., 3, 9, and 15). Three factor distributions were used to distinguish the groups. The first factor distribution was *N* ~ (0, 1), groups 1, 4, 7, 10, 13, 16, 19, 22, 25, and 28 have this factor distribution. The second factor distribution was *N* ~ (0.3, 1.5), groups 2, 5, 8, 11, 14, 17, 20, 23, 26, and 29 have this factor distribution. The third factor distribution was *N* ~ (1, 1.2), groups 3, 6, 9, 12, 15, 18, 21, 24, 27, and 30 have this factor distribution. These factor distributions are in line with the previous simulation study (Asparouhov and Muthén, 2014). In the study of Flake and McCoach (2018), they investigated three magnitudes of noninvariance, the simulated differences in loadings were: small = −0.10, medium = −0.25, and large = −0.40. For the thresholds the differences were: small = −0.20, medium = −0.50, and large = −0.80. Making some small modifications to the values used in the study of Flake and McCoach (2018), the current study had two magnitudes of noninvariance (i.e., large: unstandardized noninvariant factor loadings 0.8 ± 0.4. The standardized values were 0.63 ± 0.37 for the first group type, 0.70 ± 0.44 for the second group type, 0.66 ± 0.40 for the third group type. Noninvariant intercepts ±0.8. Small: unstandardized noninvariant factor loadings 0.8 ± 0.2. The standardized values were 0.63 ± 0.20 for the first group type, 0.70 ± 0.24 for the second group type, 0.66 ± 0.21 for the third group type. Noninvariant intercepts ±0.4). We manipulated three noninvariance rates (i.e., 0, 10, and 20%). The 20% noninvariant parameters consist of 10% noninvariant intercepts+10% noninvariant factor loadings. The 10% noninvariant parameters consist of 6.6% noninvariant intercepts+3.3% noninvariant factor loadings. The analysis began with a group size of 100, and each time that size was increased or decreased by 50 according to the accuracy of the parameter estimates, until the minimal group size required for alignment was found. To determine the power and accuracy of the parameter estimates, these four standards were adopted (Muthén and Muthén, 2002; Asparouhov and Muthén, 2014; Wang and Wang, 2019): first, the correlations between the population factor means and the estimated alignment factor means computed over groups and averaged over replications should be above 0.98; second, the absolute bias, namely the deviation between the population value and the estimated value, should not exceed 0.05 absolute value nor 10% of the population value for any parameter in the model; third, the coverage rate, namely the proportion of replications for which the 95% confidence interval contains the population parameter value, should remain between 91 and 98% for any parameter in the model; fourth, the ratios of average standard error to standard deviation should range from 0.85 to 1.15. Once these four conditions were satisfied, the average group size was chosen to keep the power close to 0.80. The value of 0.80 was used because it is a commonly accepted value for sufficient power (Muthén and Muthén, 2002). For the purpose of obtaining stable parameter estimates, 500 replications were generated for each simulation condition.

### Results of Study 1

Study 1 investigated the minimal group sizes required for alignment when the amounts of groups, the magnitudes of noninvariance and the noninvariance rates are different. Based on the different magnitudes of noninvariance, noninvariance rates, and differences in amount of groups, 15 simulation conditions were used in this study.^1^ Table 3 presents the minimal group sizes required for alignment under these 15 simulation conditions. For more detailed estimation results, please see Supplementary Table 9. The correlations between the population factor means and the estimated alignment factor means computed over groups and averaged over replications were always above 0.98 so we could conclude that alignment could produce reliable factor mean rankings. When the model had no noninvariant parameters, the minimal group size required for alignment was 250 when the amount of groups was three, the minimal group size was 150 when the amount of groups was nine, and 200 when the amount of groups was 15. When there were noninvariant parameters in the model, with all the two magnitudes of noninvariance (e.g., large, small) and all the two noninvariance rates (e.g., 10, 20%), the minimal group size required for alignment was 350 when the amount of groups was 3, and it was 250 when the amount of groups was 15. The minimal group size was 150 when the magnitude of noninvariance was large and amount of groups was nine, or when the magnitude of noninvariance was small, noninvariance rate was 10%, the amount of groups was nine; the minimal group size was 200 when the magnitude of noninvariance was small, the amount of groups was nine, but the noninvariance rate was 20%.

**Table 3:** Minimal group sizes required for alignment when magnitudes of noninvariance and noninvariance rates are different.

M	NR	g	*N*_min_
–	0%	3	250
–	0%	9	150
–	0%	15	200
Large	10%	3	350
Large	10%	9	150
Large	10%	15	250
Large	20%	3	350
Large	20%	9	150
Large	20%	15	250
Small	10%	3	350
Small	10%	9	150
Small	10%	15	250
Small	20%	3	350
Small	20%	9	200
Small	20%	15	250

In this table, M refers to magnitude of noninvariance; NR refers to noninvariance rate; g refers to amount of groups, *N*_min_ refers to minimal group size required for alignment.

## Study 2: Comparisons Among Multiple-Group CFA, Multiple-Group ESEM and Traditional Alignment When Cross-Loadings Are Included in the Model

### Research Design of Study 2

The study 2 attempted to investigate the influence of different magnitudes and numbers of cross-loading indicators on alignment optimization, make comparisons between multiple-group CFA, multiple-group ESEM, and alignment. Multiple-group ESEM is an extension of multiple-group CFA in that it also tries to establish the scalar invariance of the model and make the comparison of factor means feasible. The difference between these two models is that ESEM is able to include cross-loadings in the model itself. Multiple-group ESEM has better model fit than multiple-group CFA in most situations because neglecting the estimation of a trivial cross-loading could lead to a substantial inflated estimate of the principal factor loading (Asparouhov and Muthén, 2009). For more detailed formulation of ESEM, please see the paper of Asparouhov and Muthén which proposed ESEM for the first time in 2009. The elementary simulation conditions were identical to those used in study 1 except for the inclusion of cross-loadings and the exclusion of noninvariant parameters. Because there were no noninvariant parameters in the models, multiple-group CFA and multiple-group ESEM were directly fitted based on scalar invariance instead of gradually constraining the configural invariance, metric invariance, and scalar invariance. However, the alignment model was based on configural invariance.

Because the focus of study 2 was the influence of cross-loadings on alignment, the group size, and the noninvariance rate should be excluded. Thus, the group size was fixed to 1,000, and the noninvariance rate was fixed to 0%. The principal factor loadings were fixed to 0.8, which is a typical value in used factor analysis. There are three amounts of groups (i.e., 3, 9, and 15). The cross-loadings had two magnitudes (small: 0.1; medium: 0.3), and two proportions of indicators having cross-loading were considered (i.e., each factor had one indicator, representing 20% of the indicators; or each factor had two indicators, representing 40% of the indicators). The other simulation conditions were in line with those used in study 1.

Here are the four factor loading matrices investigated. Factor loading matrix Λ1 investigated the condition when there are 20% indicators having small-sized cross-loadings. Factor loading matrix Λ2 investigated the condition when there are 20% indicators having medium-sized cross-loadings. Λ3 investigated the condition when there are 40% indicators having small-sized cross-loadings. Λ4 investigated the condition when there are 40% indicators having medium-sized cross-loadings.


$$\Lambda 1=\left(\begin{array}{ccc} 0.8 & 0.1 & 0\\ 0.8 & 0 & 0\\ 0.8 & 0 & 0\\ 0.8 & 0 & 0\\ 0.8 & 0 & 0\\ 0.1 & 0.8 & 0\\ 0 & 0.8 & 0\\ 0 & 0.8 & 0\\ 0 & 0.8 & 0\\ 0 & 0.8 & 0\\ 0.1 & 0.1 & 0.8\\ 0 & 0 & 0.8\\ 0 & 0 & 0.8\\ 0 & 0 & 0.8\\ 0 & 0 & 0.8 \end{array}\right)\;\Lambda 2=\left(\begin{array}{ccc} 0.8 & 0.3 & 0\\ 0.8 & 0 & 0\\ 0.8 & 0 & 0\\ 0.8 & 0 & 0\\ 0.8 & 0 & 0\\ 0.3 & 0.8 & 0\\ 0 & 0.8 & 0\\ 0 & 0.8 & 0\\ 0 & 0.8 & 0\\ 0 & 0.8 & 0\\ 0.3 & 0.3 & 0.8\\ 0 & 0 & 0.8\\ 0 & 0 & 0.8\\ 0 & 0 & 0.8\\ 0 & 0 & 0.8 \end{array}\right)$$

$$\Lambda 3=\left(\begin{array}{ccc} 0.8 & 0.1 & 0\\ 0.8 & 0.1 & 0\\ 0.8 & 0 & 0\\ 0.8 & 0 & 0\\ 0.8 & 0 & 0\\ 0.1 & 0.8 & 0\\ 0 & 0.8 & 0.1\\ 0 & 0.8 & 0\\ 0 & 0.8 & 0\\ 0 & 0.8 & 0\\ 0.1 & 0.1 & 0.8\\ 0.1 & 0.1 & 0.8\\ 0 & 0 & 0.8\\ 0 & 0 & 0.8\\ 0 & 0 & 0.8 \end{array}\right)\;\Lambda 4=\left(\begin{array}{ccc} 0.8 & 0.3 & 0\\ 0.8 & 0.3 & 0\\ 0.8 & 0 & 0\\ 0.8 & 0 & 0\\ 0.8 & 0 & 0\\ 0.3 & 0.8 & 0\\ 0 & 0.8 & 0.3\\ 0 & 0.8 & 0\\ 0 & 0.8 & 0\\ 0 & 0.8 & 0\\ 0.3 & 0.3 & 0.8\\ 0.3 & 0.3 & 0.8\\ 0 & 0 & 0.8\\ 0 & 0 & 0.8\\ 0 & 0 & 0.8 \end{array}\right)$$

To determine the accuracy of the parameter estimates, the four standards in Study 1 were still adopted (Muthén and Muthén, 2002; Asparouhov and Muthén, 2014; Wang and Wang, 2019). For the purpose of obtaining stable parameter estimates, 500 replications were generated for each simulation condition.

### Results of Study 2

In study 2, the performance of multiple-group CFA, multiple-group ESEM, and alignment were investigated when there were different proportions and magnitudes of cross-loading. Because the other manipulated factors that influence parameter estimates should be excluded, the noninvariant parameters were not set in these models. Based on the different models and factor loading matrices, there were 36 simulation conditions. Tables 4–7 present some of the representative parameter estimates. These parameters include the first and third factor means of Group 2, *F*_1,2_, *F*_3,2,_ and the second and third factor means of Group 3, *F*_2,3_, *F*_3,3_. Four factor means were chosen because the alignment methodology was created for facilitating the factor mean comparisons and the accuracy of factor mean estimates was of primary interest for researchers. The first factor loading of the first factor in Group 1, *λ*_1,1,1_, and the eleventh factor loading of the third factor in Group 2, *λ*_11,3,2_ are presented because they are factor loadings with cross-loadings. The accuracy of their estimates reflects the effectiveness of the methodology being examined. The factor covariance between the first and second factor in Group 3, Ψ_12,3_ is also presented because neglecting the estimation of cross-loadings has led to inflated factor covariances in previous studies, and the factor covariance estimates of these three methodologies should be compared.

**Table 4:** Parameter estimates, coverage rates and ratios of average standard error to standard deviation of multiple-group CFA, multiple-group ESEM and alignment when the factor loading matrix is Λ1 (*N*_g_ = 1,000).

Model	g	*F*_1,2_	*F*_3,2_	*F*_2,3_	*F*_3,3_	*λ*_1,1,1_	*λ*_11,3,2_	Ψ_12,3_
Population value		0.3	0.3	1	1	0.8	0.8	0.5
MG-CFA	3	0.30 (0.95) 1.03	0.31 (0.96) 1.06	1.01 (0.95) 1.01	1.03 (0.93) 1.00	**0.86** (**0.47**) 0.98	**0.92** (**0.02**) 1.00	0.55 (**0.87**) 0.97
MG-ESEM	3	0.30 (0.95) 1.03	0.30 (0.96) 1.06	1.00 (0.95) 1.01	1.01 (0.94) 1.01	0.80 (0.96) 1.02	0.81 (0.94) 1.00	0.51 (0.95) 0.97
Alignment	3	0.30 (0.95) 1.04	0.30 (0.95) 1.07	1.01 (0.96) 1.03	1.02 (0.94) 1.03	**0.87** (**0.66**) 0.99	**0.89** (**0.38**) 1.02	0.55 (**0.87**) 0.97
MG-CFA	9	0.31 (0.95) 1.03	0.31 (0.95) 1.02	1.01 (0.95) 1.01	1.03 (0.93) 1.00	**0.86** (**0.39**) 1.08	**0.92** (**0.01**) 1.06	0.55 (**0.87**) 0.99
MG-ESEM	9	0.30 (0.95) 1.03	0.31 (0.95) 1.02	1.00 (0.95) 1.00	1.02 (0.94) 1.00	0.80 (0.95) 1.05	0.81 (0.95) 1.03	0.51 (0.95) 0.99
Alignment	9	0.30 (0.95) 1.04	0.30 (0.95) 1.03	1.01 (0.96) 1.02	1.02 (0.94) 1.02	**0.86** (**0.67**) 1.01	**0.89** (**0.34**) 1.01	0.55 (**0.87**) 0.99
MG-CFA	15	0.30 (0.95) 1.01	0.31 (0.95) 1.05	1.01 (0.94) 0.98	1.03 (0.94) 1.01	**0.86** (**0.35**) 1.01	**0.92** (**0**) 1.02	0.55 (**0.89**) 0.99
MG-ESEM	15	0.30 (0.95) 0.99	0.30 (0.96) 1.03	1.00 (0.94) 0.97	1.01 (0.94) 1.00	0.80 (0.94) 0.99	0.81 (0.94) 1.00	0.51 (0.95) 1.00
Alignment	15	0.29 (0.95) 1.01	0.30 (0.96) 1.04	1.00 (0.95) 0.99	1.01 (0.94) 1.02	**0.86** (**0.66**) 0.94	**0.89** (**0.34**) 1.01	0.55 (**0.89**) 0.99

The values inside parentheses are coverage rates, the values on the left side of the parentheses are parameter estimates, the values under the parameter estimates and coverage rates are ratios of average standard error to standard deviation. The values in bold do not meet the four standards which determine the accuracy of parameter estimates proposed in research design section.

**Table 5:** Parameter estimates, coverage rates and ratios of average standard error to standard deviation of multiple-group CFA, multiple-group ESEM and alignment when the factor loading matrix is Λ2 (*N*_g_ = 1,000).

Model	g	*F*_1,2_	*F*_3,2_	*F*_2,3_	*F*_3,3_	*λ*_1,1,1_	*λ*_11,3,2_	Ψ_12,3_
Population value		0.3	0.3	1	1	0.8	0.8	0.5
MG-CFA	3	0.31 (0.96) 1.03	0.33 (0.94) 1.05	1.04 (**0.90**) 1.00	**1.09** (**0.67**) 0.99	**0.99** (**0**) 0.98	**1.18** (**0**) 0.97	**0.66** (**0.22**) 0.98
MG-ESEM	3	0.30 (0.96) 1.03	0.31 (0.96) 1.05	1.00 (0.96) 1.00	1.02 (0.93) 1.00	80 (0.96) 1.01	0.82 (0.90) 0.85	0.51 (0.95) 0.98
Alignment	3	0.31 (0.95) 1.04	0.32 (0.96) 1.05	1.03 (0.93) 1.02	**1.06** (**0.86**) 1.01	**1.00** (**0**) 0.98	**1.07** (**0**) 0.92	**0.66** (**0.23**) 0.98
MG-CFA	9	0.31 (0.94) 1.03	0.33 (0.93) 1.01	1.04 (0.91) 1.02	**1.09** (**0.65**) 0.99	**0.99** (**0**) 1.06	**1.19** (**0**) 1.03	**0.66** (**0.20**) 0.98
MG-ESEM	9	0.30 (0.95) 1.03	0.31 (0.95) 1.02	1.00 (0.96) 1.03	1.02 (0.93) 0.99	0.80 (0.96) 1.06	0.82 (0.93) 0.94	0.51 (0.96) 1.00
Alignment	9	0.31 (0.95) 1.04	0.32 (0.96) 1.03	1.03 (0.95) 1.03	**1.06** (**0.86**) 1.00	**1.00** (**0**) 1.01	**1.07** (**0**) 0.91	**0.66** (**0.21**) 0.98
MG-CFA	15	0.31 (0.94) 1.01	0.33 (0.94) 1.05	1.04 (0.90) 0.98	**1.09** (**0.69**) 1.00	**0.99** (**0**) 0.98	**1.19** (**0**) 0.97	**0.66** (**0.19**) 0.99
MG-ESEM	15	0.30 (0.95) 1.02	0.31 (0.96) 1.05	1.00 (0.95) 0.97	1.02 (0.94) 1.00	0.80 (0.94) 1.00	0.82 (0.90) 0.95	0.51 (0.95) 0.99
Alignment	15	0.30 (0.94) **0.78**	0.31 (0.95) 1.02	1.02 (0.95) 0.98	1.05 (**0.88**) 0.99	**1.00** (**0**) 0.92	**1.07** (**0**) 0.88	**0.67** (**0.20**) **0.77**

The values inside parentheses are coverage rates, the values on the left side of the parentheses are parameter estimates, the values under the parameter estimates and coverage rates are ratios of average standard error to standard deviation. The values in bold do not meet the four standards which determine the accuracy of parameter estimates proposed in research design section.

**Table 6:** Parameter Estimates, coverage rates and ratios of average standard error to standard deviation of multiple-group CFA, multiple-group ESEM and alignment when the factor loading matrix is Λ3 (*N*_g_ = 1,000).

Model	g	*F*_1,2_	*F*_3,2_	*F*_2,3_	*F*_3,3_	*λ*_1,1,1_	*λ*_11,3,2_	Ψ_12,3_
Population value		0.3	0.3	1	1	0.8	0.8	0.5
MG-CFA	3	0.31 (0.95) 1.03	0.31 (0.96) 1.05	1.03 (0.94) 1.01	1.05 (**0.88**) 1.00	**0.86** (**0.48**) 0.98	**0.92** (**0.02**) 1.00	**0.58** (**0.73**) 0.97
MG-ESEM	3	0.30 (0.96) 1.03	0.31 (0.96) 1.05	1.00 (0.95) 1.01	1.04 (0.91) 1.02	0.81 (0.96) 1.02	0.84 (**0.85**) 1.06	0.53 (0.93) 0.98
Alignment	3	0.31 (0.95) 1.04	0.31 (0.95) 1.06	1.02 (0.95) 1.03	1.04 (0.91) 1.03	**0.86** (**0.67**) 0.98	**0.90** (**0.26**) 1.02	**0.58** (**0.74**) 0.98
MG-CFA	9	0.31 (0.95) 1.03	0.32 (0.95) 1.02	1.03 (0.93) 1.01	1.05 (**0.88**) 1.00	**0.86** (**0.39**) 1.07	**0.92** (**0**) 1.06	**0.58** (**0.74**) 0.99
MG-ESEM	9	0.31 (0.95) 1.03	0.32 (0.95) 1.02	1.00 (0.95) 1.02	1.04 (**0.90**) 0.98	0.81 (0.96) 1.08	0.84 (**0.71**) 1.04	0.53 (0.95) 1.00
Alignment	9	0.30 (0.95) 1.04	0.31 (0.96) 1.02	1.02 (95) 1.02	1.04 (0.91) 1.01	**0.86** (**0.68**) 1.01	**0.90** (**0.23**) 1.02	**0.58** (**0.75**) 0.99
MG-CFA	15	0.30 (0.94) 1.00	0.31 (0.95) 1.04	1.02 (0.94) 0.98	1.05 (**0.89**) 1.01	**0.86** (**0.35**) 1.00	**0.92** (**0**) 1.01	**0.58** (**0.72**) 1.00
MG-ESEM	15	0.30 (0.95) 1.01	0.31 (0.95) 1.03	1.00 (0.93) 0.96	1.04 (0.91) 1.01	0.81 (0.94) 0.98	0.84 (**0.67**) 1.02	0.52 (0.95) 1.00
Alignment	15	0.30 (0.95) 1.01	0.31 (0.95) 1.04	1.01 (0.95) 1.00	1.03 (0.93) 1.03	**0.86** (**0.67**) 0.94	**0.90** (**0.25**) 1.01	**0.58** (**0.73**) 1.00

The values inside parentheses are coverage rates, the values on the left side of the parentheses are parameter estimates, the values under the parameter estimates and coverage rates are ratios of average standard error to standard deviation. The values in bold do not meet the four standards which determine the accuracy of parameter estimates proposed in research design section.

**Table 7:** Parameter estimates, coverage rates and ratios of average standard error to standard deviation of multiple-group CFA, multiple-group ESEM and alignment when the factor loading matrix is Λ4 (*N*_g_ = 1,000).

Model	g	*F*_1,2_	*F*_3,2_	*F*_2,3_	*F*_3,3_	*λ*_1,1,1_	*λ*_11,3,2_	Ψ_12,3_
Population value		0.3	0.3	1	1	0.8	0.8	0.5
MG-CFA	3	0.32 (0.94) 1.03	**0.34** (**0.90**) 1.04	**1.08** (**0.75**) 1.00	**1.14** (**0.36**) 0.99	**1.00** (**0**) 0.98	**1.20** (**0**) 0.98	**0.73** (**0.02**) 0.97
MG-ESEM	3	0.31 (0.96) 1.04	0.33 (0.93) 1.02	1.00 (0.94) 1.00	**1.11** (**0.55**) 0.99	0.82 (**0.90**) **0.74**	**0.97** (**0.01**) 0.94	0.53 (**0.90**) **0.78**
Alignment	3	0.32 (0.94) 1.04	0.33 (0.93) 1.03	**1.07** (**0.83**) 1.02	**1.12** (**0.57**) 1.02	**1.00** (**0**) 0.98	**1.15** (**0**) 0.97	**0.74** (**0.03**) 0.99
MG-CFA	9	0.32 (0.93) 1.03	**0.34** (**0.88**) 1.01	**1.08** (**0.77**) 1.02	**1.14** (**0.33**) 0.99	**0.99** (**0**) 1.06	**1.20** (**0**) 1.04	**0.74** (**0.02**) 0.99
MG-ESEM	9	0.31 (0.96) 1.03	**0.34** (0.91) 1.00	1.00 (0.96) 1.05	**1.12** (**0.49**) 0.99	0.81 (0.96) 1.08	**0.97** (**0**) 1.01	0.52 (0.95) 1.01
Alignment	9	0.32 (0.95) 1.04	0.33 (0.93) 1.01	**1.06** (**0.84**) 1.03	**1.12** (**0.52**) 0.99	**1.00** (**0**) 1.02	**1.15** (**0**) 0.98	**0.74** (**0.02**) 1.00
MG-CFA	15	0.32 (0.93) 1.00	**0.34** (**0.90**) 1.03	**1.08** (**0.76**) 0.97	**1.14** (**0.36**) 0.99	**1.00** (**0**) 0.98	**1.20** (**0**) 0.97	**0.74** (**0.01**) 1.00
MG-ESEM	15	0.30 (0.95) 1.01	0.33 (0.92) 1.02	1.00 (0.94) 0.95	**1.11** (**0.52**) 0.98	0.81 (**0**.93) 0.99	**0.97** (**0**) 0.95	0.52 (0.95) 1.02
Alignment	15	0.31 (0.95) 1.01	0.33 (0.93) 1.02	**1.06** (**0.84**) 0.97	**1.11** (**0.59**) 1.00	**1.00** (**0**) 0.93	**1.15** (**0**) 0.94	**0.74** (**0.02**) 1.00

The values inside parentheses are coverage rates, the values on the left side of the parentheses are parameter estimates, the values under the parameter estimates and coverage rates are ratios of average standard error to standard deviation. The values in bold do not meet the four standards which determine the accuracy of parameter estimates proposed in research design section.

As is shown in Table 4 when the factor loading matrix was Λ1 , wherein each factor had 20% factor loading (1 factor loading) with small-sized cross-loading, all the three methodologies provided accurate factor mean estimates. While multiple-group ESEM provided accurate factor loading and factor covariance estimates, multiple-group CFA and alignment provided unacceptable factor loading and factor covariance estimates. The parameter estimates, coverage rates and ratios of average standard error to standard deviation did not have significant differences with different amounts of groups.

As is shown in Table 5, when the factor loading matrix was Λ2 , wherein each factor had 20% factor loading (1 factor loading) with medium-sized cross-loading, multiple-group ESEM provided accurate factor mean, factor loading and factor covariance estimates. Multiple-group CFA and alignment provided unacceptable factor mean estimates and inflated factor loading and factor covariance estimates. The parameter estimates, coverage rates and ratios of average standard error to standard deviation did not have significant differences with different amounts of groups.

As is shown in Table 6, when the factor loading matrix was Λ3 , wherein each factor had 40% factor loadings (2 factor loadings) with small-sized cross-loading, multiple-group ESEM and alignment both provided similar accurate factor mean estimates, but multiple-group CFA provided unacceptable results. Multiple-group ESEM could provide accurate factor loading and factor covariance estimates, but multiple-group CFA and alignment provided unacceptable factor loading and factor covariance estimates. The parameter estimates, coverage rates and ratios of average standard error to standard deviation did not have significant differences with different amounts of groups.

As is shown in Table 7, when the factor loading matrix was Λ4 , wherein each factor had 40% factor loadings (2 factor loadings) with medium-sized cross-loading, none of the three methodologies could provide accurate factor mean estimates. Multiple-group CFA provided unacceptable factor loading and factor covariance estimates, while multiple-group ESEM only provided accurate estimates for factor loadings with only one cross-loading and factor covariances. Factor loadings with two cross-loadings, such as *λ*_113,_ had inflated estimates. The parameter estimates, coverage rates and ratios of average standard error to standard deviation did not have significant differences with different amounts of groups.

## Discussion

After the completion of the two simulation variants, we were able to achieve deeper insight into the alignment model. Study 1 provided us with deeper insight into the minimal group sizes required for alignment under different simulation conditions. When the model has no noninvariant parameters, the alignment requires relatively lower group sizes. Explicitly, the minimal group size required for alignment was 250 when the amount of groups was three, the minimal group size was 150 when the amount of groups was nine, and 200 when the amount of groups was 15. When the model has noninvariant parameters, no matter how high the noninvariance rate is or what the magnitude of noninvariance is, the minimal group size required is 350 when the amount of groups is three; it is 250 when the amount of groups is 15; when the amount of groups is nine, the minimal group size required is at least 150 under most conditions, but is at least 200 when the magnitude of noninvariance is small, noninvariance rate is 20%, and the amount of groups is 9. These results show that the minimal group size required for alignment decreases at first as the amount of groups increases from three groups to nine groups, but increases gradually as the amount of groups increases from nine groups to 15 groups. Therefore, we may conclude that when the amount of groups is low, a group size of 350 is a safe rule of thumb. When the amount of groups is high, a group size of 250 is required for trustworthy results. The magnitude of noninvariance and the noninvariance rate do not affect the minimal group size required for alignment. The majority of previous simulation studies consider only two sample sizes (i.e., 100, 1,000) and have not investigated the minimal group sizes required for alignment. In this respect, then, the current study offers new insights and understanding in its development off the findings of previous studies.

Study 2 compared the performances of multiple-group CFA, multiple-group ESEM, and alignment when there are cross-loadings. Results show that multiple-group CFA provides accurate factor mean estimates when each factor had 20% factor loading (1 factor loading) with small-sized cross-loading. It always provides unacceptable factor loading and factor covariance estimates under all conditions. Multiple-group ESEM provides accurate factor mean estimates when the magnitude of cross-loading is small or when each factor had 20% factor loading (1 factor loading) with medium-sized cross-loading. It always provides accurate factor loading and factor covariance estimates except the condition when each factor had 40% factor loadings (2 factor loadings) with medium-sized cross-loading. Alignment provides accurate factor mean estimates when there are only small-sized cross-loadings in the model. It also always provides unacceptable factor loading and factor covariance estimates under all conditions because it is still a methodology based on CFA model. The parameter estimates, coverage rates and ratios of average standard error to standard deviation for each methodology are not influenced by varying the amount of groups. Thus, these results highlight the importance of checking whether there are cross-loadings which could have significant impact on factor mean estimates when using multiple-group CFA, multiple-group ESEM and alignment.

Just as this article is under review, Mplus released a new version 8.8 in April, 2022. Mplus 8.8 extends the alignment methodology in several important ways. First, the alignment methodology is implemented for the WLS estimators with the delta and theta parameterizations. This extension is valuable for those situations where the ML estimation is slow due to numerical integration. The second important generalization of the alignment methodology in Mplus 8.8 is the possibility of complex loading structures. Prior to Mplus 8.8, the alignment methodology was not able to include cross-loadings in the model. In real empirical data, the loading structure is usually not pure and cross-loadings are present. The results of study 2 in this article highlight the drawbacks of alignment in Mplus versions prior to Mplus 8.8 because alignment is not able to include cross-loadings in the model in these versions. Since the new 8.8 version is released, the simulation studies of Asparouhov and Muthén show that the so-called AESEM (aligned ESEM) perfectly resolves the issues raised in study 2 and thus, this AESEM is more generalizable, effective, and more practical to use (Asparouhov and Muthén, 2022). Another important generalization of the alignment methodology in Mplus 8.8 is the possibility to apply the methodology for a general structural equation model. It is now possible to estimate general SEM models with alignment, including adding covariates/factor predictors, adding direct effects from the covariates to the factor indicators, and correlating the factors with other dependent variables. In summary, these new features are important advances of alignment methodology and will help alignment gain more popularity.

Based on the results and conclusions drawn from this study, we include Table 8 which presents practical guidance to practitioners of which analytical methodologies are feasible in different contexts. To summarize, multiple-group CFA is more suitable for use when scalar invariance is established. Multiple-group ESEM works best when there are small-sized or only a few medium-sized cross-loadings in the model. Traditional alignment can allow for small-sized cross-loadings and a few noninvariant parameters in the model. AESEM integrates the advantages of alignment and ESEM, can provide accurate estimates when noninvariant parameters and cross-loadings both exist in the model. Compared to multiple-group CFA and multiple-group ESEM, the alignment methodology, especially the aligned ESEM (AESEM) performs well in more situations. This study is the first to present such results and conclusions, as no previous study has explored the impact of cross-loadings on the traditional alignment parameter estimation. As such, the conclusions and practical guidance on using alignment, aligned ESEM (AESEM), multiple-group CFA, and multiple-group ESEM are also one of the main contributions of this study.

**Table 8:** Applicability of multiple-group CFA, multiple-group ESEM, alignment and aligned ESEM.

Different conditions	No or few noninvariant parameters	Noninvariance rate ≤ 20%
No Cross-loading	MG-CFA is best	Alignment, AESEM
20% small-sized Cross-loadings	All four methods feasible	Alignment, AESEM
40% small-sized Cross-loadings	MG-ESEM, Alignment, AESEM feasible	Alignment, AESEM
20% medium-sized Cross-loadings	MG-ESEM and AESEM feasible	AESEM
40% medium-sized Cross-loadings	AESEM	AESEM

Although this study has some advantages and innovations, there are still some limitations. First, the amount of groups used in this current study may not have been large enough to conclude the applicability of alignment. When Asparouhov and Muthén first proposed alignment, they declared that alignment can deal with data sets having at most 100 groups (Asparouhov and Muthén, 2014). In this study, only three amounts of groups (i.e., 3, 9, and 15) were considered. Future studies could consider and explore the effects of using higher amounts of groups. Second, the simulation conditions were all optimal and excluded the influence of other manipulated conditions. But in real data, the ideal simulation conditions may not be met and the performance of alignment might be influenced by several manipulated factors or their interactions. Third, in study 1, the average group size of 100 is used as the starting point. Increasing or decreasing 50 each time, however, could be viewed a being too large change. Future research can explore the analysis using smaller changes in average group size to obtain more precise minimal average group sizes required for alignment.

To conclude, alignment is not universally applicable for any empirical data. The average group size, the complexity of the factor structure, the noninvariance rate of the model parameters, the estimation method and identification method used can all influence the performance of alignment. For a suitable use of alignment, researcher can regard alignment methodology as an alternative for measurement invariance analysis and compare its results with those of other methodologies. The model with best fit, providing the most accurate model parameter estimates, and maybe the most parsimonious model should be preferable. And with the advances of alignment methodology theory, we believe that alignment will gradually gain more popularity in empirical studies. The Mplus team will also strive for the better and easier implementation of alignment in new Mplus versions.

## Data Availability Statement

The datasets presented in this study can be found in online repositories. The names of the repository/repositories and accession number(s) can be found at: All the mplus syntax files of this study have already been uploaded to OSF official website. Here is the link: https://osf.io/xhr7b/.

## Author Contributions

CW designed the study and wrote the manuscript. CW and FH analyzed the data, and modified the manuscript. All authors have read and agreed to the published version of the manuscript.

## Conflict of Interest

The authors declare that the research was conducted in the absence of any commercial or financial relationships that could be construed as a potential conflict of interest.

## Publisher’s Note

All claims expressed in this article are solely those of the authors and do not necessarily represent those of their affiliated organizations, or those of the publisher, the editors and the reviewers. Any product that may be evaluated in this article, or claim that may be made by its manufacturer, is not guaranteed or endorsed by the publisher.

## Supplementary Material

The Supplementary Material for this article can be found online at: https://www.frontiersin.org/articles/10.3389/fpsyg. 2022.845721/full#supplementary-material

## Footnotes

^1^All the mplus syntax files of this study have already been uploaded to OSF official website. Here is the link: https://osf.io/xhr7b/.
