# Supplementary material for: Investigating the Applicability of Alignment—A Monte Carlo Simulation Study
Source: Front Psychol. 2022 Jun 24;13:845721. doi: 10.3389/fpsyg.2022.845721 (PMC9263979; doi:10.3389/fpsyg.2022.845721)
Supplement: Supplementary file 1 [file Table_9.docx]

**Appendix**

Table 9 presents some representative parameters’ estimation results of Study 1.

Table 9

Some Parameter Estimates, Coverage Rates and ratios of Average Standard Error to Standard Deviation of Alignment when Magnitudes of Noninvariance and Noninvariance Rates are Different

| M | NR | g | Ng | F_1,2_ | F_3,2_ | F_2,3_ | F_3,3_ | λ_51,3_ | v_3,2_ | v_14,3_ |
| --- | --- | --- | --- | --- | --- | --- | --- | --- | --- | --- |
| Population value | | |  | 0.3 | 0.3 | 1 | 1 | 0.8 | 0 | 0 |
| - | 0% | 3 | 200 | .31(.97)  1.07 | .30(.96)  1.04 | 1.01(.98)  **1.17** | 1.01(.97)  1.09 | .80(.98)  1.05 | 0(.96)  1.04 | .01(.97)  1.04 |
| - | 0% | 3 | 250 | .30(.96)  1.07 | .30(.97)  1.08 | 1.00(.96)  1.12 | 1.00(.97)  1.09 | .80(.97)  1.07 | .01(.97)  1.06 | .02(.97)  1.02 |
| - | 0% | 9 | 100 | **.25**(.97)  1.08 | **.26**(.95)  1.07 | .96(.94)  1.05 | .95(.95)  1.08 | .80(.97)  1.09 | .04(.95)  1.04 | .04(.96)  1.04 |
| - | 0% | 9 | 150 | .27(.95)  1.01 | .27(.94)  1.01 | .98(.96)  1.09 | .97(.96)  1.10 | .80(.97)  1.07 | .03(.95)  1.04 | .03(.97)  1.05 |
| - | 0% | 15 | 150 | **.26**(.94)  1.02 | **.25**(.93)  1.01 | .95(.95)  1.11 | .95(.96)  1.12 | .79(.98)  1.08 | .03(.96)  1.05 | **.05**(.96)  1.09 |
| - | 0% | 15 | 200 | .27(.95)  1.06 | .27(.96)  1.03 | .96(.95)  .95 | .96(.96)  1.12 | .79(.95)  .99 | .02(.96)  1.02 | .04(.95)  1.03 |
| Population value | | |  | 0.3 | 0.3 | 1 | 1 | 0.8 | 0.8 | 0.8 |
| large | 10% | 3 | 300 | .30(.96)  1.04 | .30(.95)  1.04 | .99(.95)  1.05 | 1.00(.96)  1.12 | .80(.97)  1.03 | 80(.98)  **1.17** | .01(.97)  1.07 |
| large | 10% | 3 | 350 | .30(.97)  1.11 | .30(.96)  1.07 | 1.00(.97)  1.10 | 1.00(.98)  1.14 | .80(.97)  1.03 | .80(.96)  1.00 | .01(.96)  1.03 |
| large | 10% | 9 | 100 | **.26**(.96)  1.06 | **.26**(.97)  1.14 | .97(.95)  1.05 | .96(.95)  **1.17** | .80(.97)  1.09 | .84(.95)  1.03 | .04(.97)  1.06 |
| large | 10% | 9 | 150 | .27(.95)  1.01 | .27(.95)  1.02 | .98(.96)  1.09 | .98(.96)  1.12 | .80(.97)  1.07 | .83(.95)  1.05 | .03(.97)  1.08 |
| large | 10% | 15 | 200 | .27(.96)  1.05 | .28(.97)  1.01 | .96(.95)  .97 | .97(.96)  **1.16** | .80(.95)  .99 | .82(.95)  1.00 | .03(.98)  1.07 |
| large | 10% | 15 | 250 | .28(.94)  .99 | .28(.96)  1.07 | .97(.94)  1.04 | .97(.96)  1.09 | .80(.96)  1.05 | .82(.95)  1.08 | .02(.96)  1.04 |
| Population value | | |  | 0.3 | 0.3 | 1 | 1 | 1.2 | 0.8 | 0.8 |
| large | 20% | 3 | 300 | .29(.96)  1.04 | .31(.96)  1.04 | 1.00(.94)  1.00 | 1.00(.97)  1.14 | 1.20(.96)  1.04 | .81(.98)  **1.16** | .81(.97)  1.10 |
| large | 20% | 3 | 350 | .30(.96)  1.08 | .31(.97)  1.09 | 1.00(.96)  1.08 | 1.00(.96)  1.15 | 1.21(.94)  1.01 | .80(.96)  1.01 | .80(.95)  1.07 |
| large | 20% | 9 | 100 | **.25**(.96)  1.05 | .29(.98)  1.18 | 1.00(.95)  1.06 | .99(.96)  **1.16** | 1.21(.96)  1.11 | .84(.95)  1.02 | .82(.94)  1.07 |
| large | 20% | 9 | 150 | .27(.94)  .99 | .29(.96)  1.02 | 1.00(.96)  1.05 | .99(.97)  1.15 | 1.20(.96)  1.06 | .83(.95)  1.03 | .82(.96)  1.11 |
| large | 20% | 15 | 200 | .27(.96)  1.06 | .30(.96)  1.00 | .99(.95)  1.03 | .98(.96)  **1.18** | 1.20(.96)  1.04 | .83(.95)  1.00 | .82(.97)  1.07 |
| large | 20% | 15 | 250 | .27(.92)  .97 | .30(.96)  1.08 | .99(.94)  1.01 | .99(.97)  1.08 | 1.20(.95)  1.04 | .82(.95)  1.07 | .81(.95)  1.04 |
| Population value | | |  | 0.3 | 0.3 | 1 | 1 | 0.8 | 0.4 | 0 |
| small | 10% | 3 | 300 | .30(.95)  1.06 | .30(.95)  1.02 | .99(.95)  1.05 | 1.00(.95)  1.09 | .80(.97)  1.03 | .40(.98)  **1.18** | .01(.97)  1.05 |
| small | 10% | 3 | 350 | .30(.97)  1.10 | .30(.96)  1.05 | 1.00(.97)  1.11 | 1.00(.96)  1.11 | .80(.97)  1.03 | .40(.95)  1.00 | .01(.96)  1.02 |
| small | 10% | 9 | 100 | **.26**(.96)  1.06 | **.26**(.96)  1.10 | .97(.95)  1.07 | .96(.94)  1.12 | .80(.97)  1.09 | .44(.95)  1.03 | .04(.96)  1.05 |
| small | 10% | 9 | 150 | .27(.95)  1.01 | .27(.95)  1.02 | .98(.96)  1.10 | .97(.96)  1.12 | .80(.97)  1.07 | .42(.95)  1.06 | .03(.97)  1.06 |
| small | 10% | 15 | 200 | .27(.96)  1.05 | .27(.96)  1.03 | .96(.95)  1.05 | .96(.96)  **1.16** | .79(.95)  .99 | .42(.95)  1.00 | .04(.97)  1.05 |
| small | 10% | 15 | 250 | .28(.94)  .99 | .27(.95)  1.04 | .97(.94)  1.04 | .97(.96)  1.07 | .80(.96)  1.04 | .42(.95)  1.08 | .03(.96)  1.02 |
| Population value | | |  | 0.3 | 0.3 | 1 | 1 | 1.01 | 0.4 | 0.4 |
| small | 20% | 3 | 300 | .30(.96)  1.05 | .30(.96)  1.04 | 1.00(.94)  1.03 | 1.00(.96)  1.10 | 1.01(.95)  1.04 | .41(.98)  **1.17** | .41(.95)  1.09 |
| small | 20% | 3 | 350 | .30(.96)  1.09 | .31(.97)  1.07 | 1.00(.96)  1.11 | 1.00(.96)  1.13 | 1.01(.93)  1.02 | .40(.95)  1.01 | .40(.96)  1.08 |
| small | 20% | 9 | 150 | .27(.94)  .99 | .28(.96)  1.03 | .99(.96)  1.05 | .98(.97)  **1.16** | 1.01(.96)  1.06 | .43(.94)  1.04 | .42(.95)  1.10 |
| small | 20% | 9 | 200 | .27(.95)  1.06 | .29(.96)  .99 | .99(.96)  1.07 | .99(.97)  1.13 | 1.00(.96)  1.05 | .42(.95)  1.02 | .41(.95)  1.04 |
| small | 20% | 15 | 200 | **.26**(.96)  1.05 | .29(.96)  1.01 | .98(.94)  1.03 | .97(.96)  **1.17** | 1.00(.95)  1.02 | .43(.95)  1.00 | .43(.95)  1.06 |
| small | 20% | 15 | 250 | .27(.93)  .98 | .29(.96)  1.04 | .99(.95)  1.02 | .98(.96)  1.06 | 1.01(.94)  1.05 | .43(.94)  1.08 | 1.00(.93)  .92 |

🞱 *In this table, M refers to magnitude of noninvariance; NR refers to noninvariance rate; g refers to amount of groups; N_g_ refers to average group size; The values inside parentheses are coverage rates, the values on the left side of the parentheses are parameter estimates, the values under the parameter estimates and coverage rates are ratios of average standard error to standard deviation. The values in bold don’t meet the four standards which determine the accuracy of parameter estimates proposed in research design section.*
